# Commentary: Evaluation of Breast Animation Deformity following Pre- and Subpectoral Direct-to-Implant Breast Reconstruction: A Randomized Controlled Trial

**DOI:** 10.1055/s-0043-1777254

**Published:** 2024-02-22

**Authors:** Diana L. Dyrberg, Camilla Bille, Vibeke Koudahl, Oke Gerke, Jens A. Sørensen, Jørn B. Thomsen

**Affiliations:** 1 Department of Plastic Surgery, Odense University Hospital, Odense/Lillebaelt Hospital, Vejle, Denmark; 2 Department of Plastic Surgery, Odense University Hospital, Odense, Denmark; 3 Department of Nuclear Medicine, Odense University Hospital, Odense, Denmark

Evaluation of Breast Animation Deformity following Pre- and Subpectoral Direct-to-Implant Breast Reconstruction: A Randomized Controlled Trial

The incidence and degree of BAD was significantly lower in women reconstructed by prepectoral direct-to-implant breast reconstruction. Unexpectedly, we found mild degrees of BAD in the prepectoral group. When assessing BAD, distortion can be challenging to discern from rippling.



